# Modulating the RPS27A/PSMD12/NF-κB pathway to control immune response in mouse brain ischemia-reperfusion injury

**DOI:** 10.1186/s10020-024-00870-3

**Published:** 2024-07-22

**Authors:** Xiaocheng Li, Ming Qiao, Yan Zhou, Yan Peng, Gang Wen, Chenchen Xie, Yamei Zhang

**Affiliations:** 1 Key Laboratory of Clinical Genetics, Affiliated Hospital of Chengdu University & College of Food and Biological Engineering, Chengdu, 610081 P. R. China; 2 Department of Critical Medicine, The People’s Hospital of Renshou County, Meishan, 620500 P. R. China; 3 Department of Radiation Protection Medicine, Faculty of Preventive Medicine, Air Force Medical University, Xi’an, 710032 P. R. China; 4 Department of Neurology, Affiliated Hospital of Chengdu University, Chengdu, 610082 P. R. China; 5 Key Laboratory of Clinical Genetics, Affiliated Hospital of Chengdu University, No. 82, North Section 2, 2nd Ring Road, Chengdu, Sichuan 610081 P. R. China

**Keywords:** RPS27A, PSMD12, NF-κB, Cerebral ischemia/Reperfusion, Microglia, Inflammatory factors, Immune cell infiltration

## Abstract

**Background:**

Investigating immune cell infiltration in the brain post-ischemia-reperfusion (I/R) injury is crucial for understanding and managing the resultant inflammatory responses. This study aims to unravel the role of the RPS27A-mediated PSMD12/NF-κB axis in controlling immune cell infiltration in the context of cerebral I/R injury.

**Methods:**

To identify genes associated with cerebral I/R injury, high-throughput sequencing was employed. The potential downstream genes were further analyzed using Gene Ontology (GO), Kyoto Encyclopedia of Genes and Genomes (KEGG), and Protein-Protein Interaction (PPI) analyses. For experimental models, primary microglia and neurons were extracted from the cortical tissues of mouse brains. An in vitro cerebral I/R injury model was established in microglia using the oxygen-glucose deprivation/reoxygenation (OGD/R) technique. In vivo models involved inducing cerebral I/R injury in mice through the middle cerebral artery occlusion (MCAO) method. These models were used to assess neurological function, immune cell infiltration, and inflammatory factor release.

**Results:**

The study identified RPS27A as a key player in cerebral I/R injury, with PSMD12 likely acting as its downstream regulator. Silencing RPS27A in OGD/R-induced microglia decreased the release of inflammatory factors and reduced neuron apoptosis. Additionally, RPS27A silencing in cerebral cortex tissues mediated the PSMD12/NF-κB axis, resulting in decreased inflammatory factor release, reduced neutrophil infiltration, and improved cerebral injury outcomes in I/R-injured mice.

**Conclusion:**

RPS27A regulates the expression of the PSMD12/NF-κB signaling axis, leading to the induction of inflammatory factors in microglial cells, promoting immune cell infiltration in brain tissue, and exacerbating brain damage in I/R mice. This study introduces novel insights and theoretical foundations for the treatment of nerve damage caused by I/R, suggesting that targeting the RPS27A and downstream PSMD12/NF-κB signaling axis for drug development could represent a new direction in I/R therapy.

**Supplementary Information:**

The online version contains supplementary material available at 10.1186/s10020-024-00870-3.

## Introduction

Ischemic stroke is a prevalent subtype of stroke that can result in severe brain damage in patients, ranging from disability to fatality (Meng et al. 2021; Virani et al. 2021). Cerebral ischemia/reperfusion (I/R) injury, usually resulting from strokes and heart attacks, is recognized as a rapid return of blood flow to the brain after a transient loss (Leech et al. 2019). Previous studies have reported that following ischemia/reperfusion (I/R), there is a substantial recruitment of immune cells to the ischemic site, exacerbating brain injury in patients (Jin et al. 2010; Macrez et al. 2011). This phenomenon is also associated with increased neuronal damage (Gao et al. 2020). Cerebral I/R can contribute to exacerbation of neuron injury (Gao et al. 2020). It has been documented that the large amount of infiltration of immune cells in the brain of I/R-injured mice is closely associated with the significant upregulation of inflammatory factor release (He et al. 2019). Neuroinflammation, the hallmark of which is microglial activation, can function as a regulator in cerebral I/R injury (Huang et al. 2022). Microglia are intrinsic immune cells in the brain that become activated in acute neurodegenerative diseases such as brain hypoxia and stroke, releasing inflammatory mediators that trigger the recruitment of immune cells into the brain (Hadjihambi et al. 2023; Kennedy et al. 2022). Cerebral I/R activates microglia as well as resident immune cells in the brain, and enables circulating immune cell infiltration into the ischemic lesions (Takeda et al. 2021). The specific mechanisms by which microglial cells regulate the expression of inflammatory factors and trigger immune infiltration in the context of ischemia/reperfusion (I/R) injury remain unclear. In this context, controlling immune cell infiltration in brain I/R injury is of significant importance. The interactions among immune cells as well as between immune cells and neurons, glial cells, and other cell types are crucial for understanding the roles and regulatory strategies of immune cells in brain ischemia/reperfusion injury. Identifying potential therapeutic targets and mechanisms of action in immune cells is essential for the occurrence and progression of brain ischemia/reperfusion injury.

The gene RPS27A functions as a crucial regulator in mRNA translation and ribosome accumulation (Yu et al. 2021). Studies have reported that RPS27A is significantly enriched in the chemokine pathway and immune responses as an immune-related gene (Xia et al. 2021; Chen et al. 2021a), it can mediate the interaction between the clearance-type renal cancer GYS1 and NF-κB (Chen et al. 2020). Interestingly, an increased expression of RPS27A has also been observed among sudden death syndrome patients (El-Kashef et al. 2015). In a previous cohort displaying neurodevelopmental phenotypes, half of the cases were found to have dysfunctional PSMD12 (Isidor et al. 2022). Noteworthy, a previous study revealed that disruption of PSMD12 leads to a comprehensive neurodevelopmental disorder (Küry et al. 2017). PSMD12 is a gene involved in NF-κB signal transduction (Lechner et al. 2021), and in the context of PSMD12 haploinsufficiency, there is often activation of NF-κB signaling and systemic inflammation (Yan et al., 2022). NF-κB protein is a key regulator of inflammation and immune response (Morgan and Liu 2011). Research indicates that treatment with Colebrookea oppositifolia extract - isoxanthohumol results in the inhibition of the NF-κB signaling pathway, thereby alleviating brain damage in Wistar rats (Viswanatha et al. 2021).

It is worth noting that the high-throughput sequencing combined with GO and KEGG enrichment analyses performed in the current study screened ribosomal protein S27A (RPS27A) as a key gene that might exert important functions in cerebral I/R injury. RPS27A is a pivotal regulator of mRNA translation and ribosome accumulation (Yu et al. 2021). RPS27A has been reported as an immune-associated gene enriched in chemokine pathway and immune response (Xia et al. 2021; Chen et al. 2021a). Intriguingly, increased expression of RPS27A was found in those with sudden infant death syndrome relative to that in the controls (El-Kashef et al. 2015).

In the present study, it was predicted based on protein-protein interaction (PPI) network that RPS27A may regulate the proteasome 26 S subunit, non-ATPase 12 (PSMD12)/nuclear factor kappa B (NF-κB) axis to affect cerebral I/R injury. The haploinsufficiency of PSMD12 was found in a previous cohort with neurodevelopmental phenotypes (Isidor et al. 2022). Notably, a previous study unveiled that disruption of PSMD12 resulted in a syndromic neurodevelopmental disease (Küry et al. 2017). It was revealed that RPS27A could intermediate the interaction between GYS1 and NF-κB in clear cell renal carcinoma (Chen et al. 2020). PSMD12 is a gene involved in NF-κB signaling (Lechner et al. 2021). Interestingly, systemic inflammation was found with activation NF-κB signaling in the context of PSMD12 haploinsufficiency (Yan et al., 2022). NF-κB proteins are transcription factors with crucial role in inflammation and immunity (Morgan and Liu 2011). Inactivated NF-κB pathway due to treatment with Colebrookea oppositifolia-isolated acteoside contributed to attenuation of cerebral injury in Wistar rats (Viswanatha et al. 2021). Accordingly, we proposed a hypothesis in the current study that RPS27A might regulate the immune cell infiltration to affect cerebral I/R injury involved with the PSMD12/NF‐κB axis.

This study confirms that RPS27A induces the expression of inflammatory factors in microglial cells by regulating the expression of the PSMD12/NF-κB signaling axis, thereby promoting immune cell infiltration in brain tissue and exacerbating brain damage in I/R mice. These findings offer new insights and theoretical basis for treating neural damage caused by I/R, suggesting that targeting RPS27A and the downstream PSMD12/NF-κB signaling axis for drug development could potentially pave the way for novel therapeutic approaches in I/R management.

## Materials and methods

### Ethical approval

The current study was performed with the approval of the Ethics Committee of Affiliated Hospital of Chengdu University. The animal experiment was conducted in accordance with the *Guide for the Care and Use of Laboratory Animals* (NO. PJ2020-055-01).

### Induction of cerebral I/R injury in mice

A total of 10 C57BL/6J male mice (weight: 2–4 g; 1 day old) and 150 male C57BL/6J mice (weight: 21–23 g; 8 week old) purchased from Chengdu Dashuo Biotechnology Co., Ltd. (Chengdu, China) were raised in separate cages in a specific-pathogen-free animal laboratory. All animal experiments in this study complied with the local principles for the management and use of laboratory animals to ensure adequate ventilation and temperature control (20–26 °C), while avoiding extreme temperatures and humidity. Lighting/dark cycles were clearly defined (typically 12 h light, 12 h dark) to align with the natural rhythm of mice. Postoperative care included sufficient monitoring, careful observation, and regular wound healing assessments. Appropriate analgesics were administered to alleviate postoperative pain. Regular behavioral observations of the experimental mice were conducted, assessing eating habits, exercise, social interactions, among others, to monitor their health and well-being.

A cerebral I/R mouse model was constructed by middle cerebral artery occlusion (MCAO) method (Li et al. 2021). Therefore, 135 mice were subjected to operation (I/R-injured mice). The mice were anesthetized by intraperitoneal injection with 1% pentobarbital sodium (40 mg/kg) and fixed on the operating table. After exposure of the neck, disinfection, and incision in the midline of neck, the skin, muscle, and subcutaneous tissue were bluntly separated successively. The right common carotid artery trunk, external and internal carotid arteries were fully exposed. The right common carotid artery was ligated in the proximal part with a 8 − 0 suture, and the external carotid artery was ligated in the distal part. A small incision was made at the distal part of the external carotid artery, and a 6 − 0 suture was inserted into the external carotid artery and then into the internal carotid artery. When the resistance of the insertion became obvious, the insertion was stopped and the length of the inserted suture was about 8–9 mm. After the end of the insertion, the suture was allowed to rest for 1 h in the mice with their body temperature maintained. The clot retrieval was performed, followed by a 15-hour reperfusion period before suturing the incision. Cerebral blood flow (CBF) in the middle cerebral artery (MCA) region was measured using a laser Doppler flowmeter (RWD, Shenzhen, China). The detector was secured above the cortex on the skull to identify a sharp decrease or complete interruption in CBF as indicators of ischemic brain region blood supply blockade, characteristic of ischemic brain injury (Li et al. 2021). Fifteen mice were assigned to the sham surgery group, where a nylon thread was only inserted into the external carotid artery without disrupting blood flow, following the same protocol as the surgical group. The mortality rate of mice during the procedure was below 10% (Figure S1).

### High-throughput sequencing

Following the establishment of an I/R model in male C57BL/6J mice, brain cortical tissue samples were obtained from three Sham groups and three I/R groups three days post-surgery. Total RNA was extracted from the six samples using the Total RNA Isolation Kit (catalog number: 12,183,555, Invitrogen, USA) and the quantification of total RNA was performed by measuring the OD values. The integrity of these total RNAs was assessed using agarose gel electrophoresis. High-quality total RNA was reverse transcribed into cDNA, and a RNA library was constructed for sequencing using the Illumina NextSeq 500 platform. The sequencing was performed with a read length configuration of paired-end 150 bp and the raw image data obtained was converted to raw reads through base calling. To ensure the quality of the raw reads, cutadapt was utilized to remove sequencing adapter sequences, filter low-quality sequences, with key parameters including “-m” (minimum remaining read length), “-q” (quality score threshold for trimming low-quality bases), and “-a” (specifying adapter sequences). The remaining reads were termed as “clean reads.” Subsequently, the sequences were aligned to the human reference genome using Hisat2 software, with parameters such as “--dta” (for subsequent transcriptome assembly and quantification), “--rna-strandness” (specifying the strand specificity of the RNA, such as FR or RF), and “-p” (specifying the number of threads for parallel processing). Gene expression quantification was then performed using R software, resulting in a gene expression matrix (Li et al. 2022a).

### Prediction of differentially expressed genes (DEGs) and functional analysis

Using the R software package “edgeR”, differential analysis was performed, with screening thresholds set as |log2FC| > 1 and *p* < 0.05. Using the R software package “clusterProfiler”, GO and KEGG analyses were performed, and the top 2000 DEGs with the smallest *p* value were input into the STRING website to obtain the PPI network, where the confidence value was set at 0.96. The bar graph of hub genes was drawn using Cytoscape software and R software package (Zhang et al. 2022).

### Isolation and identification of primary microglia and primary neurons

On postnatal day 1, male C57BL/6J mice were euthanized, their meninges were removed, and the brain cortex was gently dissociated with 0.25% pancreatin (catalog number: 25,200,072, ThermoFisher, USA) for 10 min. The digestion was terminated by adding an equal volume of DMEM medium (catalog number: 11,965,092, Gibco) containing 10% fetal bovine serum (FBS, catalog number: 16,140,089, Gibco). Cells were centrifuged at 800 rpm for 10 min at 37 °C, the supernatant was aspirated, and the cells were resuspended in a T-75 flask. After 10–12 days, microglial cells were separated from the mixed primary glial cells by shaking the flask, and the floating microglial cells were transferred to 12 or 24-well plates for approximately 48 h. After incubating the cells with primary antibodies CD11b (#53-0112-82, ThermoFisher), Neurofilament (#ab204893, 1:500, Abcam), and Iba-1 (#MA5-41239, ThermoFisher), the respective secondary antibodies with a wavelength of 488 nm (#SA5-10110, Invitrogen, 1:500) were added for fluorescent labeling, and flow cytometry was employed to confirm that microglial cells and neurons had a purity greater than 95%. The cells were cultured in DMEM medium supplemented with 10% FBS and antibiotics (100 U/mL penicillin and 100 µg/mL streptomycin, catalog number: 15,140,148, Gibco) at 37 °C in a 5% CO_2_ environment. All materials were sourced from ThermoFisher (USA) (Meng et al. 2019).

On postnatal day 1, male C57BL/6J mice were euthanized, their brain cortices were isolated, placed in pre-chilled PBS (catalog number: 10,010,023, Gibco, USA), minced, washed, and digested in 0.125% trypsin for 30 min. The digestion was halted by using DMEM/F12 medium containing FBS (catalog number: A4192001, Gibco, USA). The cell suspension was filtered, centrifuged (3000 g, 10 min), the cell pellets were collected, resuspended in DMEM/F12 complete medium, and cultured in a 37 °C, 5% CO2 cell culture incubator (Pu et al. 2022a). The next day, serum-free medium was used, and arabinocytidine (5 µg/mL, catalog number: 147-94-4, Sigma, USA) was added to prevent the growth of non-hippocampal neurons. Cells were maintained with regular medium changes every 72 h. Flow cytometry was used to determine the percentage of neurons to ensure a purity exceeding 95%. After 7–21 days of in vitro culture, the cells were ready for experimentation (Hou et al. 2021).

### Lentiviral transduction into primary microglia

Plasmids, plasmid-derived lentiviruses, RPS27A expression plasmids, RPS27A-specific shRNA plasmids and their related lentiviruses were all obtained from Hanbio Biotechnology Co., Ltd. (Shanghai, China). Cells upon 50% confluency were infected with lentivirus (10 MOI), followed by addition of 5 µg/mL of puromycin (A1113803, Gibco). The cells were cultured for 3 days to construct a stably infected cell line (Liu et al. 2019).

### Oxygen-glucose deprivation reperfusion (OGD/R) cell model

OGD/R cell model construction: mature microglia were cultured with serum-free DMEM in an OGD environment with 5% CO_2_ and 95% N_2_ at 37 °C for 2 h, followed by further culture with normal medium with 5% CO_2_ and 95% O_2_ for 24 h, which mimicked the microenvironment of cerebral I/R injury in vivo (Figure S3) (Hou et al. 2021).

Cells were used as controls, induced by OGD/R, or induced by OGD/R and further treated with RPS27A-overexpression (oe)-negative control (NC), RPS27A-oe, RPS27A-short hairpin RNA (sh)-NC or RPS27A-sh (Table S1).

### Co-culture of microglia with neurons

Primary microglia cultured under OGD for 2 h were seeded in 6-well plates (1 × 10^5^ cells/well) and primary hippocampal neurons were seeded in cell chambers (PIHP03050, Millipore Corp., Billerica, MA) (1 × 10^5^ neurons/chamber), followed by co-culture of microglia with neurons for 24 h (Zhang et al. 2021).

### MTT-based method

The neurons after co-culture were seeded in 96-well plates containing 0.5% FBS with the density of 5 × 10^3^ cells/well. The vitality of neurons was detected 24 h later using a MTT kit (C0009S, Beyotime, Shanghai, China). The absorbance at 570 nm was measured with a microplate reader (Multiskan SkyHigh, Thermo Fisher Scientific).

### Flow cytometry

Apoptosis was detected by flow cytometry using the Annexin V-FITC/PI kit (C1062L, Beyotime). Co-cultured neurons were seeded in 6-well plates with the density of 1 × 10^6^ cells/well. Cells were resuspended with 195 µL Annexin V-FITC binding solution, followed by incubation in darkness with 5 µL Annexin V/FITC and 10 µL PI for 15 min at room temperature.

### In vivo animal experiments

Mice were sham-operated, subjected to I/R, or injected with lentiviral vector expressing RPS27A-oe-NC, RPS27A-oe, RPS27A-sh-NC or RPS27A-sh *via* the lateral ventricle (40 µL) before reperfusion and then subjected to I/R (*n* = 12). At 24 h after the reperfusion, all the mice were subjected to experiments.

Each group of mice consisted of 12 individuals. Following behavioral testing and peripheral blood collection, 6 mice were euthanized by cervical dislocation for cortical tissue analysis. The remaining 6 mice underwent intraperitoneal injection of 1% pentobarbital sodium (40 mg/kg) for anesthesia, followed by cardiac perfusion with PBS and 4% paraformaldehyde (PFA). The whole brain was then collected for histological staining experiments.

To ascertain the relationship between RPS27A, PSMD12, and NF-κB, experiments were conducted on brain I/R injury mice with silenced RPS27A. Specifically, the mice were treated with a PSMD12-specific shRNA plasmid along with the NF-κB selective agonist Betulinic acid (Catalog number: S3603, Selleck Chemicals, China) and the NF-kappaB Activation inhibitor VI, a benzoxathiole compound (ab145954, Abcam, USA), to observe the impact of brain I/R injury (Bibikova et al. 2014).

Mice were used as controls, injected with lentiviral vector expressing PSMD12-sh-NC, PSMD12-sh (Table S2) or treated with 1 µM betulinic acid (1 µM), 5 µM betulinic acid (5 µM), 10 µM betulinic acid (10 µM), 5 µM benzoxathiole (ben) and then subjected to reperfusion. *n* = 12. All lentiviruses were all obtained from Hanbio Biotechnology Co., Ltd. (Shan et al. 2022).

### Neurological deficit scoring

Neurologic deficit was evaluated for the experimental animals, including a modified neurological severity score (mNSS) test, rotarod test, and forelimb grip strength test. The mNSS test is a comprehensive test of sensory function, motor function, reflexes and balance ability. The detailed scoring criteria are shown in Table S3. The higher score indicates more serious disease condition (Chen et al. 2001).

The rotarod test (IITC Life Science, Woodland Hills, CA) was used to assess motor deficits and perceptual coordination. Mice were placed on the rotarod instrument before I/R, and the rotation speed was gradually accelerated from 0 to 40 rpm. The training was performed for 3 consecutive days. After I/R, the residence time of mice on the rotarod at 40 rpm was recorded.

The forelimb grip strength test was performed using a dynamometer (Bioseb, Pinellas Park, FL). The grip meter sensor was connected to a T bar. When the mouse paw grasped the T bar, the mouse tail was grasped and the mouse was slowly pulled horizontally until it could not grasp the T bar. The grip dynamometer recorded the maximum grip strength value of the moment when the mouse leaved the T bar. The researchers who scored the mice knew nothing about the experimental grouping.

### TTC staining

Mice were anesthetized and perfused with PBS and 4% paraformaldehyde. Next, the brain was quickly removed, dissected into coronal sections and placed in the rat brain matrix. Subsequently, a 2% TTC solution (T8877, Sigma) was used for staining in darkness at 37℃ for 15 min, and the white infarct and red non-infarct areas were visible. Brain infarct area was calculated using ImageJ image processing software. The infarct volume ratio indicating the severity of I/R was presented by the ratio of total cerebral infarction volume to total brain volume. The total infarct volume of each brain was calculated based on infarction area (mm^2^) of all brain slices from the same hemisphere (Luo et al. 2021).

### Flow cytometric sorting of cells isolated from brain tissues

Mice were anesthetized and intracardially perfused with 40 mL of cold PBS, and the ischemic hemisphere (ipsilateral to I/R) was separated by DMEM containing 4.5 g/L D-glucose and 10% FBS. Single cell suspension was obtained using a manual grinder, and the mixture was filtered using a filter with a pore size of 70 μm, followed by stratifying using Percoll gradient of 30–70% (17,089,101, Cytiva, New York, NY). Gradient centrifugation was performed at 2000 rpm for 20 min. When cells appeared around the interface, they were collected and labeled using the following fluorophore-labeled primary antibodies: anti-CD45 (#103,113, 1:2000), anti-CD11b (#53-0112-82, 1:400), anti-CD4 (#MCD0405, 1:400), anti-CD8 (#A14786, 1:400), anti-Ly6G (#12-9668-82, 1:400), anti-Ly6C (#128,031, 1:400), anti-F4/80 (#123,105, 1:400), anti-B220 (#103,228, 1:400). CD45, Ly6C, F4/80, and B220 primary antibodies were all purchased from BioLegend (San Diego, CA), and CD11b, CD4, CD8, and Ly6G primary antibodies were all purchased from Invitrogen. Data were collected using the FacsDiva software of the Cytoanalyzer (BD), and the data were analyzed using the FlowJo software (v 10.5.3).

### Flow cytometric sorting of immune cells isolated from the peripheral blood

After mouse anesthesia, peripheral blood was collected from the eyes of the mice with a test tube containing EDTA (E8008, Sigma). Subsequently, 1 mL ACK lysates (A10492-01, Gibco) were added to each tube for destruction of red blood cells, and immune cells in the peripheral blood were finally obtained after centrifugation at 2000 rpm for 5 min. The cells were incubated with fluorophore-labeled primary antibodies.

### Flow cytometry for the detection of glial cell/neuron cell purity

Co-cultured glial cells/neurons were seeded into 6-well plates at a density of 1 × 10^6^ cells per well. Following cell collection, the cells were incubated with primary antibodies against Iba-1 (#MA5-41239, 1:500, Thermo Fisher Scientific, USA) and Neurofilament (#ab204893, 1:500, Abcam, USA) to label glial cells and neurons. Incubation at room temperature for two hours was followed by the addition of secondary antibodies with a wavelength of 488 nm (#SA5-10110, Invitrogen, 1:500) and further incubation in the dark at room temperature for 2 h. Cell nuclei were counterstained with DAPI dye (catalog number: 62,247, Thermo Fisher Scientific, 1:1000). Flow cytometry analysis was conducted within 20 min, and the fluorescent cell ratio was determined (Chen et al. 2021b).

### ELISA

Mouse cerebral cortex tissues or cultured microglia were collected and lysed. After centrifugation, cell supernatants were collected. TNF-α (H052-1), IFN-γ (H025), IL-1β (H002), and IL-6 (H007-1-2) were determined by ELISA according to the instruction of manufacturer’s kit (NanJing JianCheng Bioengineering Institute, Nanjing, China) (Luo et al. 2022).

### Measurement of GSH, MDA, and ROS in brain tissues

The mouse brain tissues were added with normal saline (1:9) pre-cooled at 4 °C on an ice bath. After homogenization with a high-speed homogenizer, the centrifugation was performed at 4000 r/min for 10 min. The supernatant was collected and separately stored at -20℃. GSH (A006-2-1, NanJing JianCheng Bioengineering Institute) and MDA (A003-1-2, NanJing JianCheng Bioengineering Institute) levels were measured according to the kit instructions.

ROS was tested according to the kit instruction (S0033S, Beyotime). Fresh cerebral cortical tissues (50 mg) were washed with PBS and then homogenized with 1 mL of lysis buffer-A using a homogenizer. Next, the homogenate was centrifuged at 1000 g for 10 min at 4 °C, and the supernatant was collected. The fluorescent probe (10 µmol/L) of 2,7-dichofluorescein diacetate (DCFH-DA) was then added to the supernatant (190 µL), and the samples were incubated at 37 °C in darkness for 30 min. Finally, the green fluorescence intensity was quantified with an automated fluorescence microplate reader (Thermo Fisher Scientific), with excitation wavelength of 488 nm and emission wavelength of 530 nm. The level of ROS in the tissue is the ratio of the fluorescence intensity to the protein concentration (Hu et al. 2022).

### RNA extraction and RT-qPCR

Total RNA was extracted from the microglia and cerebral cortex tissues using the TRIzol reagent (10,296,010, Thermo Fisher Scientific) in accordance with the manufacturer’s guidelines (Maurya et al. 2018). Next, 1 µg of RNA was reversely transcribed using TaqMan reverse transcription reagents (A25741, Thermo Fisher Scientific). PCR analysis was performed using the PowerUp SYBR Green Master Mix Kit (A25741, Thermo Fisher Scientific). With GAPDH as the internal reference, gene expression was quantified by 2^-ΔΔCt^ method. The list of primers is provided in Table S4.

### Western blot

Total protein were extracted from cells and cerebral cortex tissues using RIPA lysis buffer with 4% protease inhibitor (P0013B, Beyotime) and protein concentrations were quantified by BCA Protein Assay (P0010S, Beyotime). Lysates were loaded on SDS-PAGE gels for transfer to a PVDF membrane, followed by incubation with the primary antibodies including anti-RPS27A (#ab172293, Abcam, Cambridge, UK; 1:500), anti-PSMD12 (#SAB4502462, Sigma; 1:1000), anti-p-IκBα (#SAB4504445, Sigma; 1:1000), anti-IκBα (#SAB4501994, Sigma; 1:1000), anti-NF-kB p65 (#SAB5700046, Sigma; 1:1000), anti-histone H3 (#06-755, Sigma; 1:1000) and anti-GAPDH (#ab181602, Abcam; 1:10000). Following incubation with secondary antibody goat anti-rabbit (A0208, Beyotime), blots were visualized using an enhanced chemiluminescence kit (P0018FS, Beyotime) on the enhanced chemiluminescence detection system (ChemiDoc XRS+, Bio-Rad Laboratories, Hercules, CA) and quantified by ImageJ software as normalized to GAPDH.

### Immunofluorescence staining

Mice were anesthetized and then perfused with PBS and 4% paraformaldehyde. Brains were collected, soaked overnight in 4% paraformaldehyde and dehydrated in 15% and 30% glucose solution. The brains were sliced (20 μm thick) using a cryomicrotome (CryoStar NX50 HOP, Thermo Fisher Scientific), fixed in 4% paraformaldehyde for 15 min at room temperature, cleared with 0.25% Triton X-100, and blocked in sealing buffer containing 2% FBS for 2 h. The sections were incubated at 4℃ with primary antibodies including anti-Iba-1 (#MA5-41239, 1:500, Thermo Fisher Scientific), antibody against Neurofilament (#ab204893, 1:500, Abcam, USA), anti-RPS27A (#ab227011, 1:500, Abcam) and anti-Ly6G (#12-9668-82, Invitrogen).

The next day, the sections were incubated with the secondary antibody (#PA184709, Invitrogen, 1:500) with a wavelength of 570 nm at room temperature for 2 h. Nuclei were counterstained with DAPI staining (62,247, Thermo Fisher Scientific, 1:1000). Images were acquired using a fluorescence microscope (Olympus X73), and the Image-J software was used to quantify the positivity.

### Nissl staining

Mouse cerebral cortex tissues were fixed in 4% paraformaldehyde for 48 h, dehydrated, embedded, and made into paraffin sections for the Nissl staining performed according to the instructions of the Nissl staining kit (C0117, Beyotime). The sections were stained with Nissl staining solution for 3–10 min, washed with ethanol, dehydrated, cleared and sealed. The sections were observed under a microscope, and Image J software was used for quantification (Xu et al. 2018).

### TUNEL staining

Apoptosis was determined using TUNEL apoptosis detection kit (C1091, Beyotime). Paraffin-embedded sections were prepared, dehydrated, stained, and sealed according to the manufacturer’s instructions (Ya et al. 2018). The sections were observed under a light microscope, and Image J software was used for quantification (Liang et al. 2019).

### Statistical analysis

Data were analyzed by SPSS 21.0 statistical software (IBM Corp., Armonk, NY). The measurement data are expressed as mean ± standard deviation. The comparisons between the two groups were conducted using *t* test. Data between multiple groups were compared by one-way ANOVA. *p* < 0.05 demonstrated that the difference was statistically significant.

## Results

RPS27A was upregulated and inflammatory factor release was increased in the cerebral cortex tissues of I/R-injured mice.

**Fig. 1:**
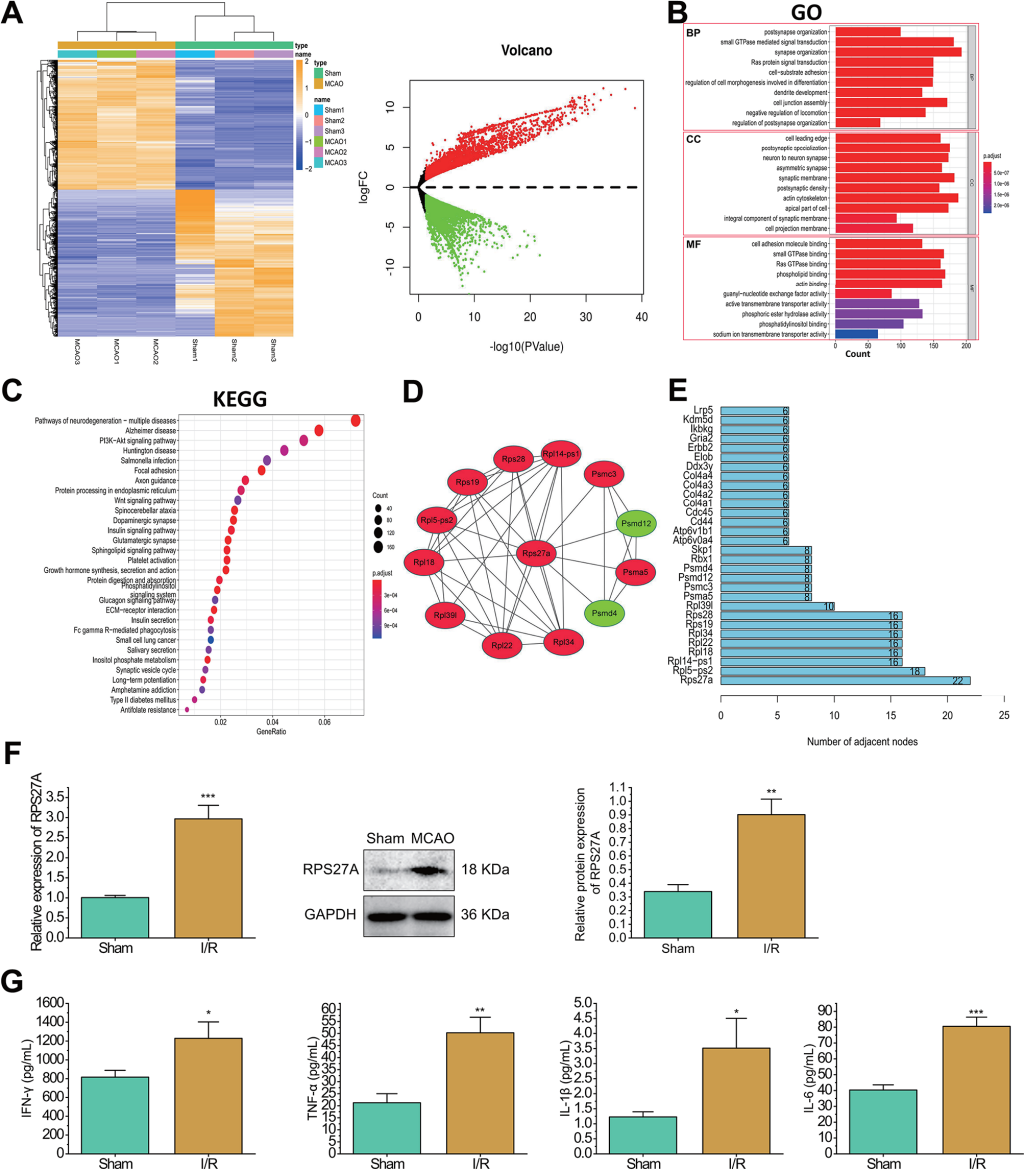
Prediction of DEGs and the expression of RPS27A and inflammatory factors in the cerebral cortex tissues of I/R-injured mice. **A**, Heat map and volcano map of the DEGs in the cerebral cortex tissues of brain I/R-injured mice detected by high-throughput sequencing. In the volcano map, red represents upregulation, and green represents downregulation. **B**, GO analysis of DEGs in the cerebral cortical tissues of I/R-injured mice. **C**, KEGG analysis of DEGs in the cerebral cortical tissues of I/R-injured mice. The color and size of the bubbles in KEGG indicate the significance of the differences and the number of enriched genes, respectively. **D**, PPI network of DEGs in the cerebral cortical tissues of I/R-injured mice. Red represents the upregulation, and green represents downregulation. **E**, Bar graph of the hub genes in the PPI network. **F**, RT-qPCR and Western blot to determine the mRNA and protein expression of RPS27A in the cerebral cortical tissues of sham-operated and I/R-injured mice. **G**, ELISA to determine the release of inflammatory factors in the cerebral cortical tissues of sham-operated and I/R-injured mice. *n* = 3. * *p* < 0.05, ** *p* < 0.01, *** *p* < 0.001 vs. the sham group

First, the behaviors of mice were observed, and the results showed that the mNSS of I/R-injured mice was significantly increased compared with that of sham-operated mice, but rotation and grasping abilities were notably decreased, indicating successful model construction (Figure S3). Subsequently, high-throughput sequencing of cerebral cortical tissue samples of sham-operated mice and I/R-injured mice yielded a total of 9222 Differential Expression Analysis (DEGs), among which 4324 genes were significantly upregulated and 4898 genes were downregulated (Fig. 1A).

Next, functional enrichment analysis was performed on the differentially expressed genes. Results from GO and KEGG analyses revealed that in synaptic tissue, the signaling transduction regulated by small GTPases and synaptic tissue ranked highest in the “biological process” category of GO. In the “cellular component” category of GO, cell polarization, post-synaptic differentiation, and interneuronal synapse ranked highest. Adhesion factor binding, small GTPase binding, and Ras GTPase binding were the top-ranked categories in the “molecular function” of GO. The top three pathways identified in KEGG were related to neurodegenerative diseases, Alzheimer’s disease, and the PI3K-Akt signaling pathway. Within the GO enrichment pathways, associations were found with neurodegenerative disease-related pathways such as post-synaptic tissue, synaptic tissue, post-synaptic differentiation, interneuronal synapse, and post-synaptic dense area. Moreover, the top two pathways in the KEGG rankings related to neurodegenerative diseases, neurodegenerative diseases, and Alzheimer’s disease were all associated with neurodegenerative diseases (Liu et al. 2022, 2023; Li et al. 2022b). These differentially expressed genes are implicated in various neurodegenerative diseases (Fig. 1B, C). Next, the top 2000 differentially expressed genes, ranked by *p*-value from lowest to highest, were submitted to the STRING website. A confidence level of 0.96 was set, and unbound proteins were excluded, resulting in 1380 proteins with mutual relationships identified. Subsequently, the “stv” file obtained from the STRING website was utilized in R programming to generate a bar chart of the Hub genes in the PPI network (Fig. 1D). Among them, the top 10 Hub genes were identified as RPS27A (having the highest degree value), RPL5-PS2, RPL14-PS1, RPL18, RPL22, RPL34, RPS19, RPS28, RPL39L, and PSMA5, all of which showed significant upregulation in the I/R group. Furthermore, the Cytoscape software was employed to visualize the differential genes associated with RPS27A in the PPI network (Fig. 1E). In addition, studies have shown that RPS27A was also abnormally upregulated in the brainstem of newborns with sudden infant death syndrome (El-Kashef et al. 2015). Therefore, we hypothesized that RPS27A might play an important role in the induction of cerebral I/R injury.

Further, RT-qPCR and Western blot found that RPS27A mRNA and protein expression was significantly upregulated in the cerebral cortex tissues of I/R-injured mice (Fig. 1F). In addition, the ELISA results showed that the inflammatory factors such as IFN-γ, TNF-α, IL-1β and IL-6 were also notably upregulated in the cerebral cortical tissues of I/R-injured mice compared with those in the sham-operated mice (Fig. 1G, Figure S3).

Taken together, RPS27A was notably upregulated in the cerebral cortex tissues of I/R-injured mice, accompanied by elevated inflammatory factor release.

### OGD/R promoted the expression of RPS27A and inflammatory factors in microglia

**Fig. 2:**
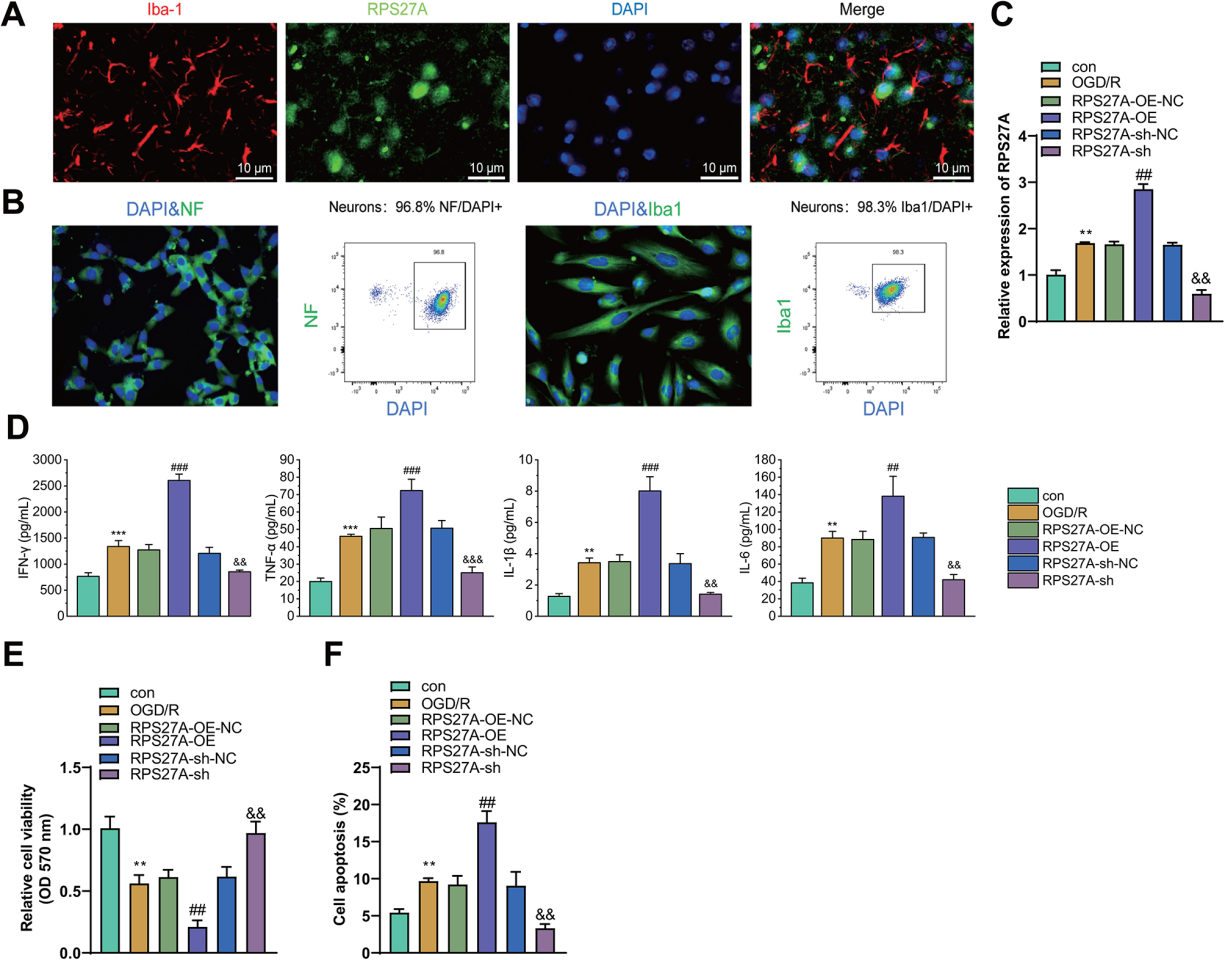
Effects of OGD/R on the expression of RPS27A and inflammatory factors in microglia. **A**, Immunofluorescence to detect the localization of RPS27A and microglia in the cerebral cortical tissues of I/R-injured mice. **B**, Flow cytometry analysis to assess the purity of primary astrocytes/neurons (> 95%). **C**, RT-qPCR to detect RPS27A mRNA expression in microglia. **D**, ELISA to determine the expression of IFN-γ, TNF-α, IL-1β and IL-6 in microglial cells. E, MTT to measure the viability of neurons after co-culture with microglia. **F**, Flow cytometry to determine the apoptosis of neurons after co-culture with microglia. All cell experiments were repeated three times. ** *p* < 0.01, *** *p* < 0.001 vs. the control group. ## *p* < 0.01, ### *p* < 0.001 vs. the RPS27A-oe-NC group. && *p* < 0.01, &&& *p* < 0.001 vs. the RPS27A-sh-NC group

The study shifted to investigate whether high expression of RPS27A in cerebral cortex tissues of I/R-injured mice was related with microglia. The immunofluorescence staining was performed on cerebral cortex tissues of the I/R-injured mice, showing that area with RPS27A overexpression in cerebral cortex tissues was highly overlapped with the area where microglia existed (Fig. 2A), indicating that RPS27A was mainly expressed in microglia.

Primary microglia and primary neurons with purity greater than 95% were obtained through primary cell culture (Fig. 2B), and mouse primary microglia with stable RPS27A overexpression or silencing through transfection (Fig. 2C). The RPS27A-sh-2 sequence with optimal silencing efficiency was used for transfection (Figure S4A), and OGD/R method was applied for simulating in vitro cerebral I/R injury. ELISA showed that the expression of IFN-γ, TNF-α, IL-1β and IL-6 was significantly increased in the OGD/R-induced microglia compared with that in the control microglia. RPS27A overexpression increased while RPS27A silencing diminished the expression of IFN-γ, TNF-α, IL-1β and IL-6 in OGD/R-induced microglia (Fig. 2D). MTT and flow cytometry showed that RPS27A silencing in OGD/R-induced microglia was beneficial to maintain the neuron viability and inhibit neuron apoptosis, whereas RPS27A overexpression resulted in the opposite effects (Fig. 2E-F).

The above results indicated that OGD/R promoted RPS27A expression in microglia, while RPS27A silencing in microglia favored neuron survival and suppressed neuron apoptosis, and its effect was related to the inhibition of inflammatory factor release by microglia.

### Silencing of RPS27A ameliorated cerebral injury in the I/R-injured mice

**Fig. 3:**
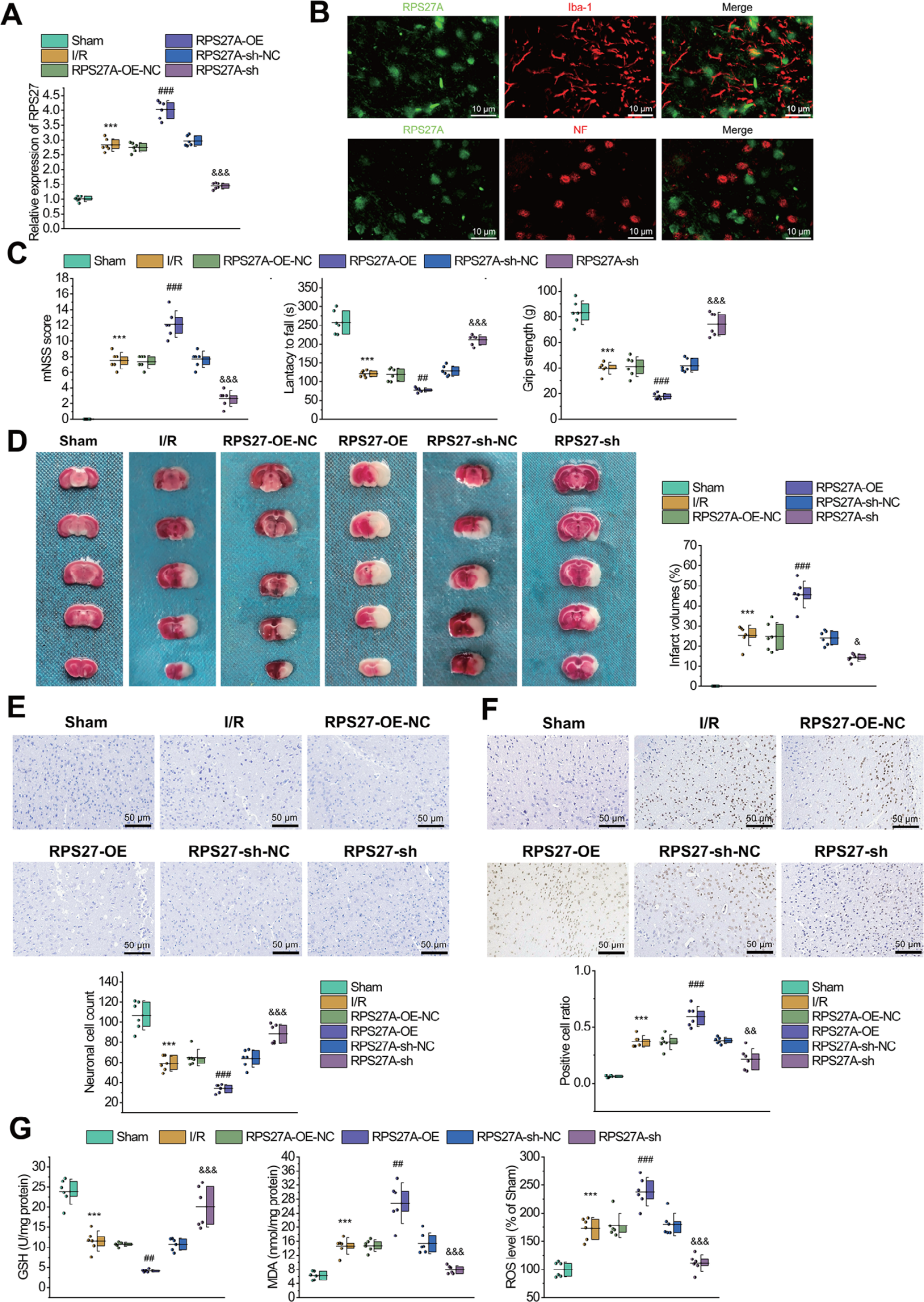
Improvement of I/R-induced Brain Damage by RPS27A Silencing in Mice. **A**, RT-qPCR to measure the mRNA expression of RPS27A in the cerebral cortical tissues of sham-operated mice and I/R-injured mice with overexpression or silencing of RPS27A. **B**: Immunofluorescence detection of the subcellular localization of RPS27A in transfected cells, microglial cells, and neurons. **C**, Behavioral evaluation of neurological injury of sham-operated mice and I/R-injured mice with overexpression or silencing of RPS27A. **D**, TTC staining to detect area of cerebral infarction in sham-operated mice and I/R-injured mice with overexpression or silencing of RPS27A. **E**, Nissl staining to detect neuron injury in cerebral cortical tissues of sham-operated mice and I/R-injured mice with overexpression or silencing of RPS27A. **F**, TUNEL staining to detect neuron apoptosis in the cerebral cortical tissues of sham-operated mice and I/R-injured mice with overexpression or silencing of RPS27A. **G**, Measurement of GSH, MDA and ROS production in the cerebral cortical tissues of sham-operated mice and I/R-injured mice with overexpression or silencing of RPS27A. *n* = 6. *** *p* < 0.001 vs. the sham-operated mice. ## *p* < 0.01, ### *p* < 0.001 vs. the RPS27A-oe-NC group. & *p* < 0.05, && *p* < 0.01, &&& *p* < 0.001 vs. the RPS27A-sh-NC group

To further explore the effect of RPS27A on cerebral I/R injury in vivo, we constructed a mouse model of cerebral I/R injury (Fig. 3A) with RPS27A overexpressing or silencing. Neurofilament and Iba1 were used to label neurons and microglial cells, respectively. Following transfection, the expression of RPS27A was examined in the cells, revealing a co-localization signal only in microglial cells, with no associated expression observed in neurons (Fig. 3B). The results of behavioral assessments showed that the neurological function of I/R-injured mice was significantly impaired compared with sham-operated mice. RPS27A silencing improved neurological functions of I/R-injured mice, whereas the overexpression of RPS27A aggravated the neurological injury of I/R-injured mice (Fig. 3C).

Meanwhile, TTC staining showed that cerebral infarct area was increased significantly in I/R-injured mice compared with that in sham-operated mice. Cerebral infarct area was notably decreased in I/R-injured mice with RPS27A silencing, while the effect was opposite in the presence of RPS27A overexpression (Fig. 3D). Nissl staining and TUNEL staining also revealed that Nissl bodies in neurons of I/R-injured mice were significantly decreased compared with those in sham-operated mice, indicating increased injury and apoptosis of neurons. The injury and apoptosis of neurons were notably decreased in I/R-injured mice with RPS27A silencing, while the effect was opposite in response to RPS27A overexpression (Fig. 3E-F).

In addition, the levels of MDA and ROS were significantly increased whereas the level of GSH was notably decreased in the cerebral cortex tissues of I/R-injured mice than in sham-operated mice. RPS27A silencing notably downregulated MDA and ROS production and upregulated GSH level in the cerebral cortex tissues of I/R-injured mice, while RPS27A overexpression could contribute to opposite effects (Fig. 3G).

The aforementioned results suggested that silencing of RPS27A could alleviate cerebral injury in the I/R-injured mice.

### RPS27A silencing inhibited immune cell infiltration by reducing the release of inflammatory factors in I/R-injured mice

**Fig. 4:**
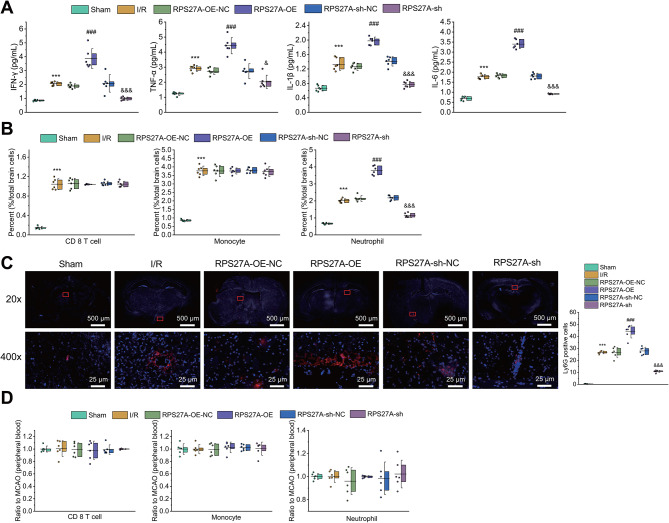
Effects of RPS27A on inflammatory factors in the brain of I/R-injured mice. **A**, ELISA to detect release of inflammatory factors in cerebral cortical tissues of I/R-injured mice in response to overexpression or silencing of RPS27A. **B**, Flow cytometry to determine immune cells in ischemic hemisphere of I/R-injured mice in response to overexpression or silencing of RPS27A. **C**, Immunofluorescence staining to measure number and distribution of neutrophils (indicated by red) in the whole brain of I/R-injured mice in response to overexpression or silencing of RPS27A. **D**, Flow cytometry to determine immune cells in peripheral blood collected from eyeballs of I/R-injured mice in response to overexpression or silencing of RPS27A. *n* = 6. ** *p* < 0.01 vs. the sham group. ## *p* < 0.01 vs. the RPS27A-oe-NC group. & *p* < 0.05, &&& *p* < 0.001 vs. the RPS27A-sh-NC group

To further explore the effect of RPS27A on the release of inflammatory factors in the I/R-injured mice, we examined the changes of IFN-γ, TNF-α, IL-1β and IL-6 expression. Based on ELISA results, the release of inflammatory factors was notably increased in the cerebral cortical tissues of I/R-injured mice than in that of sham-operated mice. RPS27A silencing decreased the inflammatory factor release in the cerebral cortical tissues of I/R-injured mice, while RPS27A overexpression brought about an opposite effect (Fig. 4A).

To further investigate the effect of RPS27A on immune cell infiltration of whole brain in I/R-injured mice, we examined immune cell changes in the mouse ischemic hemisphere. Flow cytometry showed that the content of CD8 T cells, monocytes, and neutrophils was notably increased in the ischemic hemisphere tissues of I/R-injured mice compared with that in sham-operated mice. RPS27A silencing had no effect on the content of CD8 T cells and monocytes in brain of I/R-injured mice, whereas it alleviated infiltration of neutrophils; however, infiltration of neutrophils could be aggravated by RPS27A overexpression (Fig. 4B). In addition, immunofluorescence staining also displayed that RPS27A silencing reduced whereas RPS27A overexpression increased the number of neutrophils in the ischemic hemisphere tissues of I/R-injured mice (Fig. 4C).

For exploring whether RPS27A reduced the entry of neutrophils into the ischemic hemisphere by reducing the proportion of immune cells in the peripheral blood, we collected the blood of the eyeball from I/R-injured mice and measured the content of peripheral immune cells by flow cytometry. The results showed that RPS27A overexpression or silencing did not cause significant change in CD8 T cells, monocytes and neutrophils in peripheral blood (Fig. 4D), indicating that RPS27A did not affect immune cells in peripheral blood.

The aforementioned results indicated that RPS27A silencing could reduce the release of inflammatory factors in I/R-injured mice by inhibiting immune cell infiltration.

RPS27A silencing reduced immune cell infiltration and cerebral injury in the brain of I/R-injured mice by regulating the PSMD12/NF-κB axis.

**Fig. 5:**
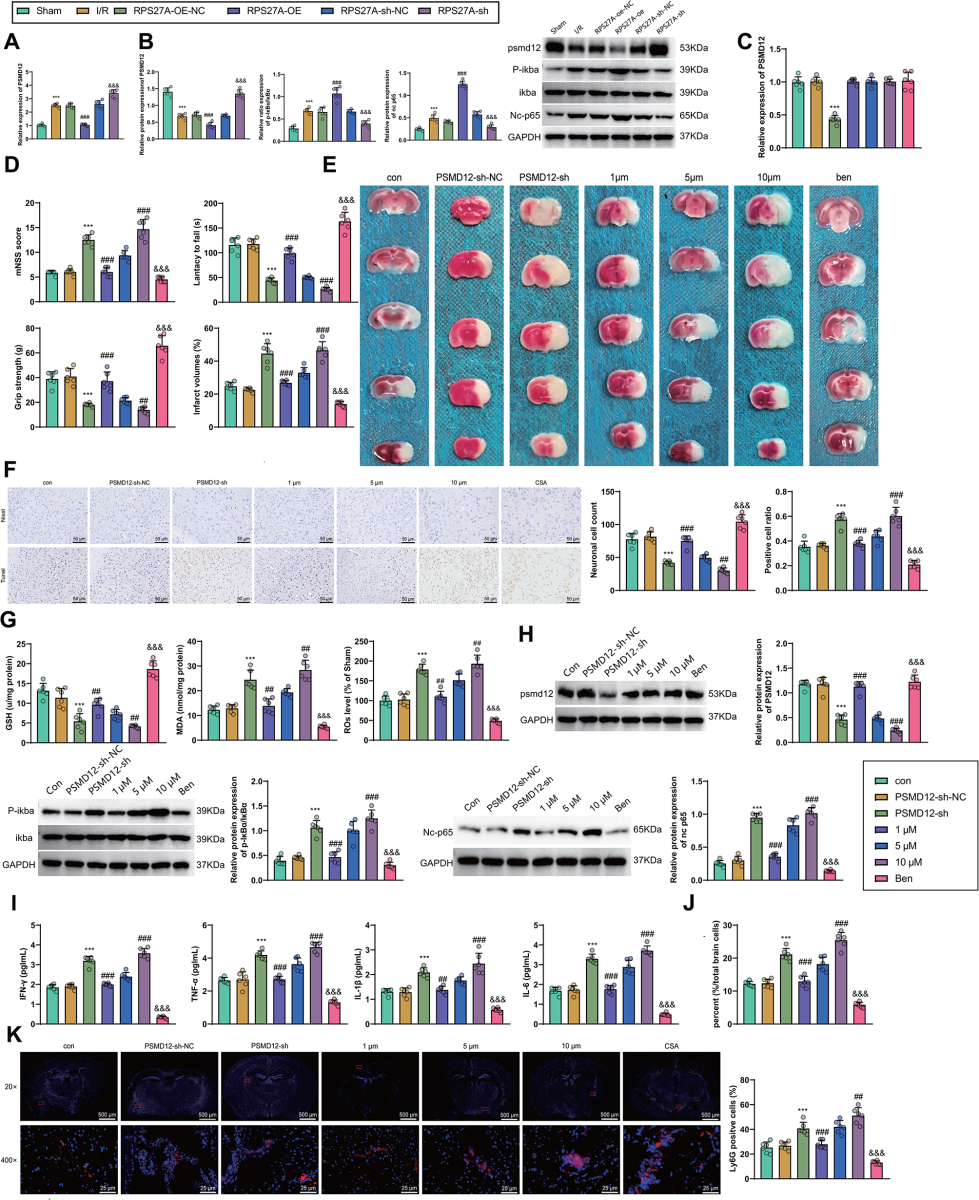
The effects of the selective activator/inhibitor of PSMD12 and NF-κB, Betulinic acid/benzoxathiole, on the silenced RPS27A in I/R mice **A**, The mRNA expression of PSMD12 in the cerebral cortical tissues of I/R-injured mice in response to overexpression or silencing of RPS27A as determined by RT-qPCR. **B**, The protein expression of PSMD12, IκBα and NC-p65 and the phosphorylation level of IκBα in the cerebral cortical tissues of I/R-injured mice in response to overexpression or silencing of RPS27A as determined by Western blot. **C**, RT-qPCR to measure the mRNA expression of PSMD12 in the cerebral cortical tissues of the I/R-injured mice in response to silenced PSMD12 or betulinic acid. **D**, Behavioral evaluation of cerebral injury in the I/R-injured mice in response to silenced PSMD12 or betulinic acid. **E**, TTC staining to detect the cerebral infarct size of I/R-injured mice in response to silenced PSMD12 or betulinic acid. **F**, Nissl and TUNEL staining to detect the neuron cell injury and apoptosis in I/R-injured mice in response to silenced PSMD12 or betulinic acid, respectively. **G**, Detection of the levels of GSH, MDA and ROS in the cerebral cortical tissues of the I/R-injured mice in response to silenced PSMD12 or betulinic acid. **H**, The protein expression of PSMD12, IκBα and NC-p65 and the phosphorylation level of IκBα in the cerebral cortical tissues of mice in response to silenced PSMD12 or betulinic acid as determined by Western blot. **I**, ELISA to measure the release of inflammatory factors in the cerebral cortical tissues of I/R-injured mice in response to silenced PSMD12 or betulinic acid. **J**, The content of neutrophils in the ischemic hemispheres of I/R-injured mice in response to silenced PSMD12 or betulinic acid as determined by flow cytometry. **K**, Immunofluorescence staining to detect the number and distribution of neutrophils in the whole brain of I/R-injured mice in response to silenced PSMD12 or betulinic acid. *n* = 6. * *p* < 0.05, ** *p* < 0.01, *** *p* < 0.001 vs. the sham group or control group. ## *p* < 0.01 vs. the RPS27A-oe-NC group. && *p* < 0.01 vs. the RPS27A-sh-NC group

Evidence exists reporting that PSMD12 deficiency upregulated the expression of TNF-α, IL-1β, and IL-6 through NF-κB signaling, while the activation of NF-κB was also observed in I/R-induced microglia (Yan et al., 2022; Pu et al. 2022b). In addition, the aforementioned PPI analysis illustrated a connection between RPS27A and PSMD12 (Fig. 1D). Therefore, we examined the expression of PSMD12, the phosphorylation level of IκBα and the nuclear NF-κB p65 content in the brain cerebral cortex tissues of mice with stable RPS27A overexpression or silencing. The RT-qPCR and Western blot results showed that the mRNA and protein expression of PSMD12 was notably decreased whereas the phosphorylation level of IκBα and the nuclear NF-κB p65 content were increased in cerebral cortical tissues of I/R-injured mice with RPS27A overexpression; RPS27A silencing could result in opposite effects (Fig. 5A, B).

To further investigate the significance of the PSMD12/NF-κB signaling axis in the regulation of immune cell infiltration by RPS27A, we silenced PSMD12 using the highly efficient sh1 sequence (Figure S4B), and observed the effects of the NF-κB selective agonist Betulinic acid and the inhibitor benzoxathiole on brain I/R injury in mice. The results revealed that silencing PSMD12 inhibited the expression of PSMD12 in the cerebral cortical tissues of the brain I/R injury mice in the control group, whereas Betulinic acid and benzoxathiole had no effect on the expression of PSMD12 (Fig. 5C). Furthermore, silencing PSMD12 or treatment with Betulinic acid intensified behavioral changes in the control group of mice with brain I/R injury, increased the infarct size in the brain and oxidative stress levels in the cortical tissue, and promoted apoptosis of brain neurons. In contrast, administration of benzoxathiole decreased behavioral changes in mice with brain I/R injury, reduced the infarct size and oxidative stress levels in the cortical tissue, while also lowering apoptosis of brain neuron cells (Fig. 5D-G).

Moreover, Western blot experiments revealed that silencing PSMD12 or treating with Betulinic acid both promoted phosphorylation of IκBα and increased nuclear NF-κB p65 content in the cortical tissue of mice with control brain I/R injury, whereas administration of benzoxathiole decreased phosphorylation of IKBA and nuclear NFKB p65 content in mouse cortical tissue (Fig. 5H). ELISA assays indicated that silencing PSMD12 or using Betulinic acid upregulated the expression levels of IFN-γ, TNF-α, IL-1β, and IL-6 in the cortical tissue of mice with control brain I/R injury, while benzoxathiole lowered the expression levels of these inflammatory factors (Fig. 5I). Flow cytometry and immunofluorescence results demonstrated that silencing PSMD12 or using Betulinic acid reversed the decrease in neutrophil infiltration in the brain tissue of control I/R mice (Fig. 5J, K).

These results suggest that alterations in RPS27A expression can impact downstream changes in PSMD12 mRNA and protein expression levels and variations in nuclear NF-κB p65 content. Silencing PSMD12 inhibited PSMD12 expression in the cortical tissue of mice with control brain I/R injury, while Betulinic acid had no effect on PSMD12 expression. Additionally, silencing PSMD12 or using Betulinic acid both increased nuclear NF-κB p65 content in the cortical tissue of mice with control brain I/R injury. Conversely, administration of benzoxathiole yielded results opposite to the NF-κB activator Betulinic acid, alleviating symptoms in I/R-injured mice while reducing phosphorylation of IκBα and the expression levels of associated inflammatory factors in the cortical tissue. Therefore, RPS27A may regulate the NF-κB signaling pathway downstream through PSMD12, promoting the secretion of inflammatory factors by microglial cells and exacerbating neutrophil infiltration and brain damage in I/R mice.

## Discussion

Ischemia/reperfusion (I/R) injury in the brain is a cardiovascular disease characterized by the activation of intrinsic immune responses and the production of inflammatory cytokines within the brain. In this study, we reported a potential molecular mechanism by which RPS27A may affect brain I/R injury. In this mechanism, RPS27A could mediate the PSMD12/NF-κB axis, promoting the secretion of inflammatory factors in microglial cells and the infiltration of immune cells into the brain I/R injury environment. Changes in the expression level of RPS27A were found to impact downstream alterations in PSMD12, with NF-κB being downstream of PSMD12 and regulated by it. Elevated levels of RPS27A were observed in mice with brain I/R injury, accompanied by increased release of inflammatory factors. Furthermore, following OGD/R, the expression of RPS27A in brain microglial cells was enhanced, while silencing RPS27A in microglial cells could potentially benefit neuronal survival by inhibiting neurodegeneration through suppressing the release of inflammatory factors. Understanding the interactions among immune cells, as well as between immune cells and neurons, glial cells, and other cells, and exploring potential therapeutic targets for immune cells in cerebral ischemia/reperfusion injury are crucial for the occurrence and development of brain damage. This finding offers new insights and theoretical basis for treating nerve injuries caused by I/R: RPS27A and the PSMD12/NF-κB axis could potentially serve as new therapeutic targets for regulating the occurrence and development of inflammatory reactions in cerebral ischemia/reperfusion injury. This signaling pathway may aid in the development of new drugs or interventions to alleviate or prevent immune infiltration into brain tissue, thus reducing the severity of brain damage. Additionally, developing new diagnostic methods to evaluate the severity of brain injuries in patients through monitoring the expression levels of inflammatory factors in brain tissue is crucial. Previous studies did not report a direct functional connection between RPS27A and PSMD12; however, we observed this unique interaction in I/R mice, and whether this interaction exists in other immune-inflammatory diseases remains to be investigated. Our research group currently lacks other mouse models of immune diseases, and further studies on whether other genes are involved in the PSMD12-RPS27A regulatory pathway are ongoing, which were not extensively addressed in this study but will be the focus of our future research. Moreover, studying how RPS27A specifically affects the activation of microglial cells and the inflammatory response in the context of cerebral ischemia/reperfusion injury will be a focus of our subsequent research. Studies have reported that RPS27A interacts with Cxcl3 to regulate the activation of Il-18 in microglial cells, which may have similar disease mechanisms to I/R (Khayer et al. 2020).

It is worth noting that there is limited research on the interaction between RPS27A/PSMD12 and NF-κB. Interestingly, previous studies have found that RPS27A mediates the interaction between GYS1 and NF-κB, promoting the phosphorylation and nuclear entry of p65 in clear cell renal cell carcinoma. Additionally, in 18 psoriasis patients with a good response to methotrexate treatment, upregulated RPS27A protein participated in the activation of NF-κB. Previous studies have reported that after I/R, a large number of immune cells are recruited to the ischemic site, exacerbating brain damage in patients, leading to aggravated neuronal injury. Significant infiltration of immune cells and increased release of inflammatory factors in the brain of I/R-damaged mice are closely related. Microglial cells and resident immune cells in the brain are activated by brain I/R, with circulating immune cells infiltrating the ischemic lesion. However, there is limited research on how microglial cells regulate the expression of inflammatory factors and induce immune infiltration in I/R-induced brain damage. This study found that RPS27A activates the NF-κB signaling pathway through the PSMD12/NF-κB axis, promoting the expression of inflammatory factors in microglial cells, leading to immune infiltration of brain tissue, ultimately exacerbating brain injury in mice following I/R. The report mentioned earlier shows the regulatory relationship between RPS27A/PSMD12 and NF-κB. Interestingly, a previous study found that the intermediate product of RPS27A and the interaction between GYS1 and NF-κB promoted the phosphorylation and nuclear translocation of p65 in renal clear cell carcinoma (Chen et al. 2020).

Accumulated evidence indicates the involvement of the NF-κB pathway in the development of brain I/R injury. For instance, PNGL has been shown to reduce the total NF-κB and NF-κB phosphorylation levels in ischemic brain, thereby aiding in the suppression of HMGB1-induced inflammation, while also inhibiting the activation of microglial cells in the hippocampus and cortex. Furthermore, extracellular vesicles derived from dental pulp stem cells exhibit anti-inflammatory effects through the inhibition of the NF-κB pathway, thus ameliorating brain I/R injury. Treatment with rhFGF21, by inhibiting NF-κB, helps alleviate neuroinflammation mediated by microglia/macrophages and reduces immune cell infiltration following brain I/R. In this study, the role of RPS27A in brain I/R injury is mediated through the regulation of the PSMD12/NF-κB pathway.

To ensure biological reproducibility (*N* ≥ 3) and to mitigate the impact of individual variability on the effective screening of key targets, we selected three rats as experimental samples in some experiments such as RNAseq. Moreover, considering the cost implications of high-throughput sequencing, using *n* = 3 as the sample size per group is common practice in high-throughput RNA-seq experiments (Zhang et al. 2023, 2024; Li et al. 2024). However, due to the limited number of sequenced mouse samples, the experimental results need further validation through an increase in the number of sequenced samples or verification in human brain I/R injury samples. Additionally, to minimize the influence of confounding factors like sex hormones on the consistency of experimental results, only male mice were chosen for this study, disregarding potential misconceptions in the screening of treatment methods or deepening the understanding of disease mechanisms that may arise from gender differences. Subsequent research will use female mice as a model to enhance the credibility of our findings. The sham-operated mice used in this study do not undergo actual ischemia-reperfusion injury, therefore, utilizing ischemic mice as a control in future studies will better mimic clinical conditions.

## Conclusion

**Fig. 6:**
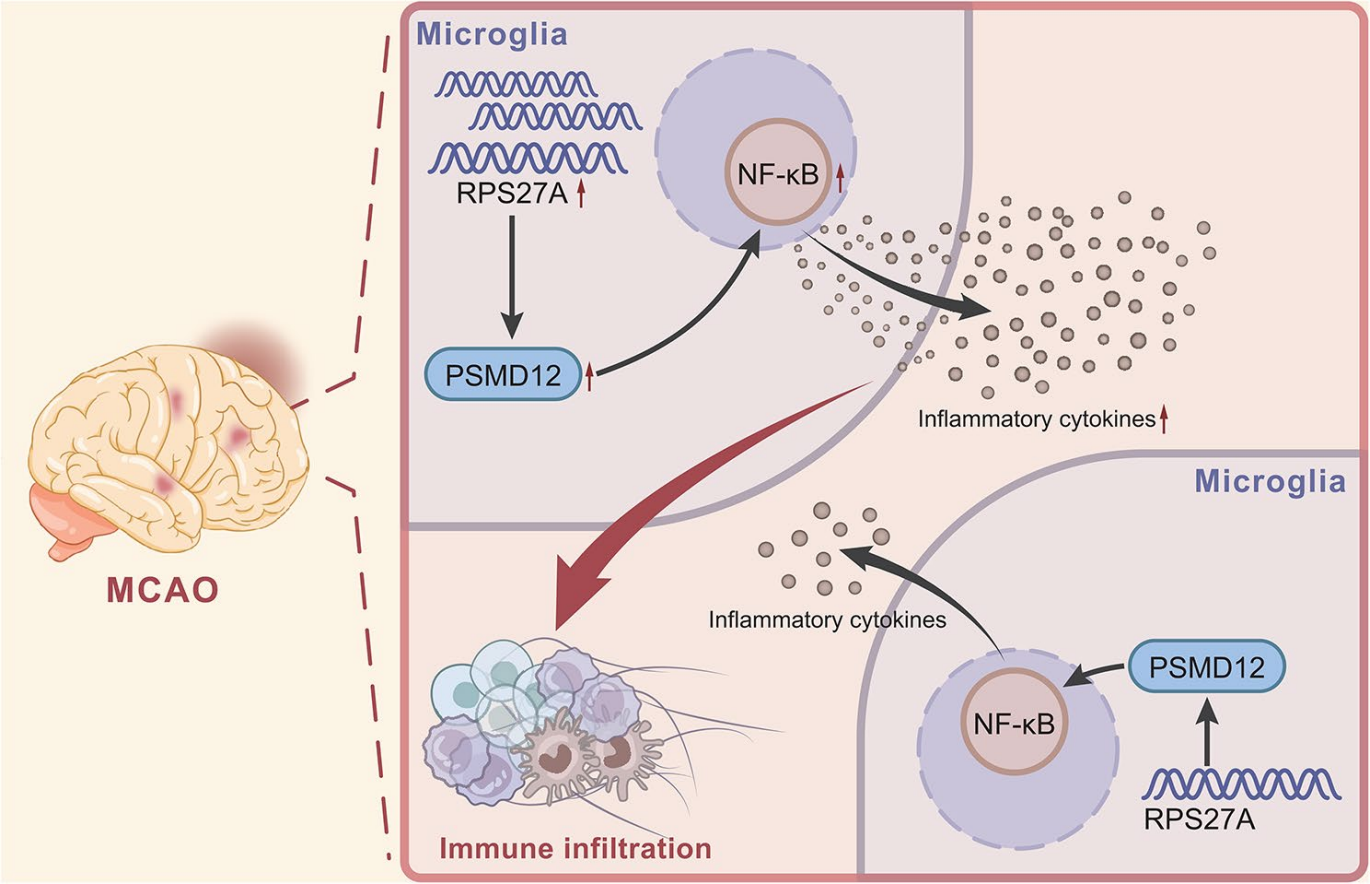
Schematic illustration of the molecular mechanism of RPS27A for inflammatory factor secretion by microglia and immune infiltration in cerebral I/R injury. RPS27A activates the NF-κB pathway through the PSMD12/NF-κB axis, thereby promoting the release of inflammatory factors in microglia, which induces immune infiltration in I/R-injured brain tissues and finally exacerbates cerebral I/R injury in mice

Taken together, the present study provides evidence indicating that RPS27A might activate the NF-κB pathway through the PSMD12/NF-κB axis, which promoted the release of inflammatory factors in microglia and induced immune infiltration in brain tissues of mice in response to cerebral I/R injury (Fig. 6). This study may provide a new idea for the alleviation of cerebral I/R injury. However, considering the small number of sequenced mouse samples, the experimental results need to be verified with a larger sample size or in human cerebral I/R injury samples.

### Electronic supplementary material

Below is the link to the electronic supplementary material.

Supplementary Material 1

Supplementary Material 2

## Data Availability

The datasets generated and/or analyzed during the current study are available from the corresponding author on reasonable request.

## Abbreviations

I/R: Ischemia/Reperfusion
GO: Gene Ontology
KEGG: Kyoto Encyclopedia of Genes and Genomes
PPI: Protein-Protein Interaction
RPS27A: Ribosomal Protein S27A
PSMD12: Proteasome 26 S Subunit, Non-ATPase 12
NF-κB: Nuclear Factor Kappa B
OGD/R: Oxygen-Glucose Deprivation/Reoxygenation
MCAO: Middle Cerebral Artery Occlusion
DEGs: Differentially Expressed Genes
ELISA: Enzyme-Linked Immunosorbent Assay
GSH: Glutathione
MDA: Malondialdehyde
ROS: Reactive Oxygen Species
RT-qPCR: Real-Time Quantitative Polymerase Chain Reaction
TTC: 2,3,5-Triphenyltetrazolium Chloride
mNSS: Modified Neurological Severity Score
NC: Negative Control
sh: Short Hairpin
oe: Overexpression
MOI: Multiplicity of Infection
MAP2: Microtubule-Associated Protein 2
DAPI: 4’,6-diamidino-2-phenylindole
TUNEL: Terminal deoxynucleotidyl Transferase dUTP Nick End Labeling
SD: Standard Deviation
ANOVA: Analysis of Variance
PBS: Phosphate-Buffered Saline
FBS: Fetal Bovine Serum
DMEM: Dulbecco’s Modified Eagle Medium
PI: Propidium Iodide
FITC: Fluorescein Isothiocyanate
ACK: Ammonium-Chloride-Potassium
EDTA: Ethylenediaminetetraacetic Acid
BCA: Bicinchoninic Acid
PVDF: Polyvinylidene Difluoride
GAPDH: Glyceraldehyde 3-Phosphate Dehydrogenase
SDS-PAGE: Sodium Dodecyl Sulfate Polyacrylamide Gel Electrophoresis
