# Supplementary material for: Modulating the RPS27A/PSMD12/NF-κB pathway to control immune response in mouse brain ischemia-reperfusion injury
Source: Mol Med. 2024 Jul 22;30:106. doi: 10.1186/s10020-024-00870-3 (PMC11265174; doi:10.1186/s10020-024-00870-3)
Supplement: Supplementary file 1 — Supplementary Material 1 [file 10020_2024_870_MOESM1_ESM.doc]

**Table S1** shRNA sequences of RPS27A

| shRNA | Sequence (5’-3’) |
| --- | --- |
| RPS27A-sh-NC | 5’-CCGGACTAAGTCTTTGACTAC-3’ (Sigma) |
| RPS27A-sh-1 | 5’-CCGGACTTTGTCTGACTACAA-3’ (Sigma) |
| RPS27A-sh-2 | 5’-GCATAAGAGGAAGAAGGTTAA-3’ (Sigma) |

Note: RPS27A, ribosomal protein S27A; sh-, shRNA, short hairpin RNA-; NC, negative control.

**Table S2** shRNA sequences of PSMD12

| shRNA | Sequence (5’-3’) |
| --- | --- |
| PSMD12-sh-NC | 5’-GCTGGAAGAAATTAAATACCC-3’ (Sigma) |
| PSMD12-sh-1 | 5’-GCTGGAAGAAATTCCCAAATA-3’ (Sigma) |
| PSMD12-sh-2 | 5’-CATTCGCACACAGATCATTAG-3’ (Sigma) |

Note: PSMD12, proteasome 26S subunit, non-ATPase 12; sh-, short hairpin RNA-; NC, negative control.

**Table S3** Criteria of mNSS

| Tests | Score |
| --- | --- |
| Motor tests | 6 |
| Raising the mouse by tail | 3 |
| Forelimb flexion | 1 |
| Hindlimb flexion | 1 |
| Head moved 10° to the vertical axis within 30 s | 1 |
| Placing the mouse on floor (normal = 0; maximum = 3) | 3 |
| Normal walk | 0 |
| Inability to walk straight | 1 |
| Circling to one side | 2 |
| Falling down to one side | 3 |
| Sensory tests | 2 |
| Placing test (visual and tactile) | 1 |
| Proprioceptive test (deep sensation, pushing paw against table edge to stimulate the muscles of the limbs) | 1 |
| Beam balance tests (normal = 0; maximum = 6) | 6 |
| Balances with stable posture | 0 |
| Grasps one side of the beam | 1 |
| Hugs the beam, with 1 limb falling down from the beam | 2 |
| Hugs the beam, with 2 limbs falling down from the beam, or spinning on the beam (> 60 s) | 3 |
| Attempts to balance on the beam but falls off (> 40 s) | 4 |
| Attempts to balance on the beam but falls off (> 20 s) | 5 |
| Falls off, without attempting to balance or hang on the beam (< 20 s) | 6 |
| Reflex absence and abnormal movements | 4 |
| Pinna reflex (head shake when auditory meatus is touched) | 1 |
| Corneal reflex (eye blink when cornea is lightly touched with cotton) | 1 |
| Startle reflex (motor response to a brief noise from snapping a clipboard paper) | 1 |
| Seizures, myoclonus, hypotonia | 1 |
| Maximum points | 18 |

Note: 1-6, mild injury; 7-12, moderate injury; 13-18, severe injury.

**Table S4** Primer sequences for RT-qPCR

| Gene | Sequence (5’-3’) |
| --- | --- |
| RPS27A (mouse) | Forward: 5’-GCCATCGTGGGTGAGTGTAT-3’ |
|  | Reverse: 5’-TAGATCCGACGACACCCGT-3’ |
| PSMD12 (mouse) | Forward: 5’-CGAGGGGTCCTATCTGTCCA-3’ |
|  | Reverse: 5’-CCTGCTGCCACTTGTCACTT-3’ |
| GAPDH (mouse) | Forward: 5’-GGGTCCCAGCTTAGGTTCAT-3’ |
|  | Reverse: 5’-AATCCGTTCACACCGACCTT-3’ |

Note: RT-qPCR, real-time reverse transcription polymerase chain reaction; RPS27A, ribosomal protein S27A; PSMD12, proteasome 26S subunit, non-ATPase 12; GAPDH, glyceraldehyde-3-phosphate dehydrogenase.
