# Supplementary material for: Modulating the RPS27A/PSMD12/NF-κB pathway to control immune response in mouse brain ischemia-reperfusion injury
Source: Mol Med. 2024 Jul 22;30:106. doi: 10.1186/s10020-024-00870-3 (PMC11265174; doi:10.1186/s10020-024-00870-3)
Supplement: Supplementary file 2 — Supplementary Material 2 [file 10020_2024_870_MOESM2_ESM.docx]

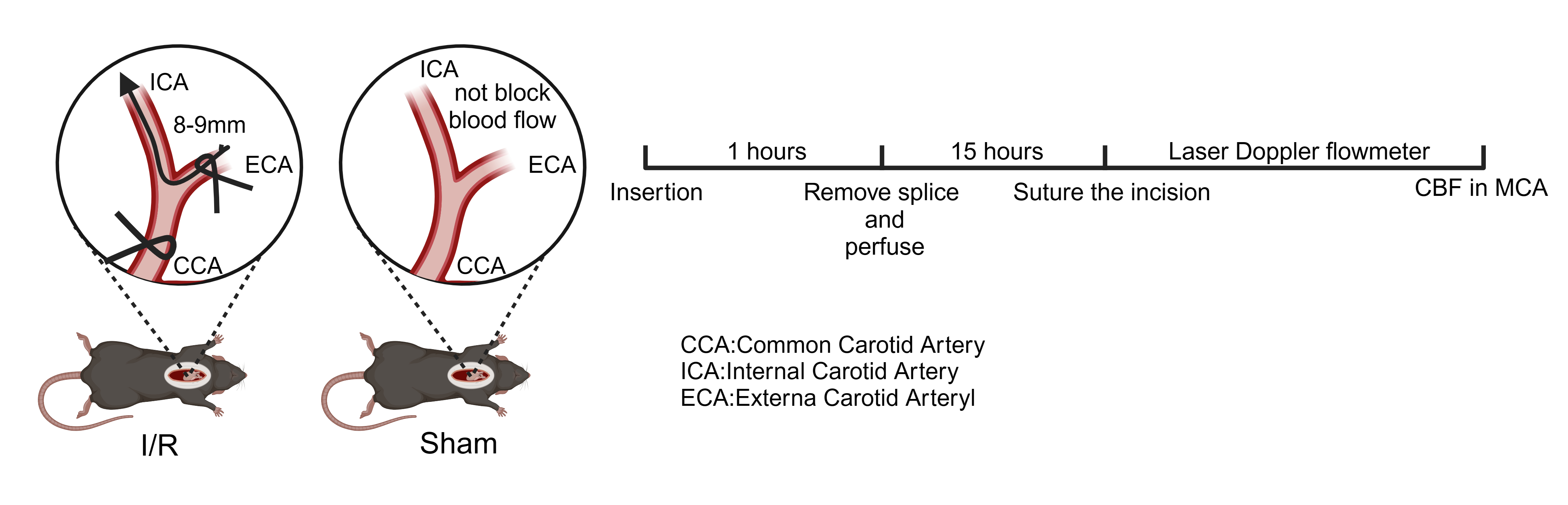


**Figure S1** Schematic illustration of the construction of the cerebral ischemia-reperfusion (I/R) injury mouse model.


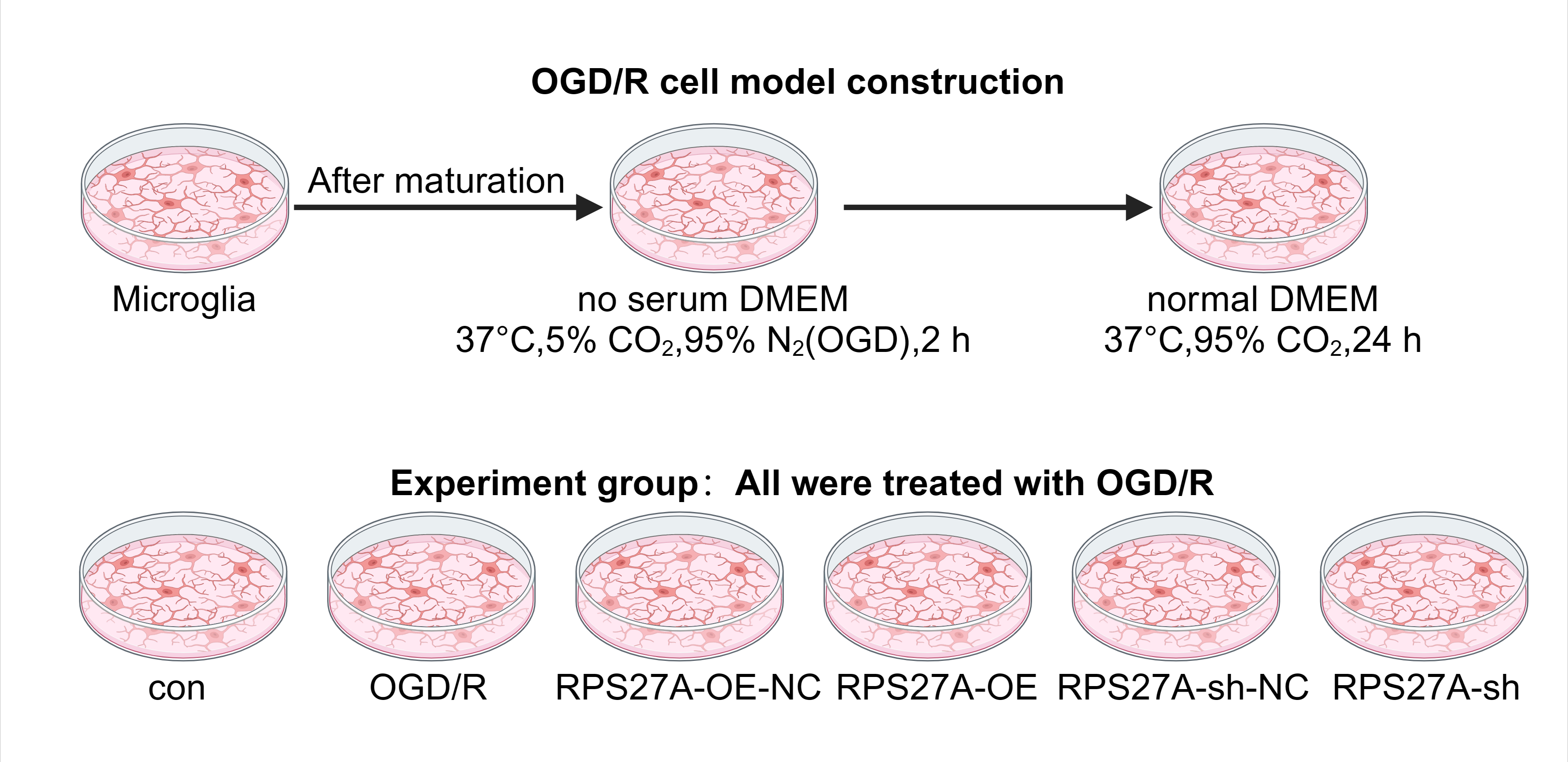


**Figure S2.** Schematic diagram of oxygen-glucose deprivation/reperfusion (OGD/R) cell model.


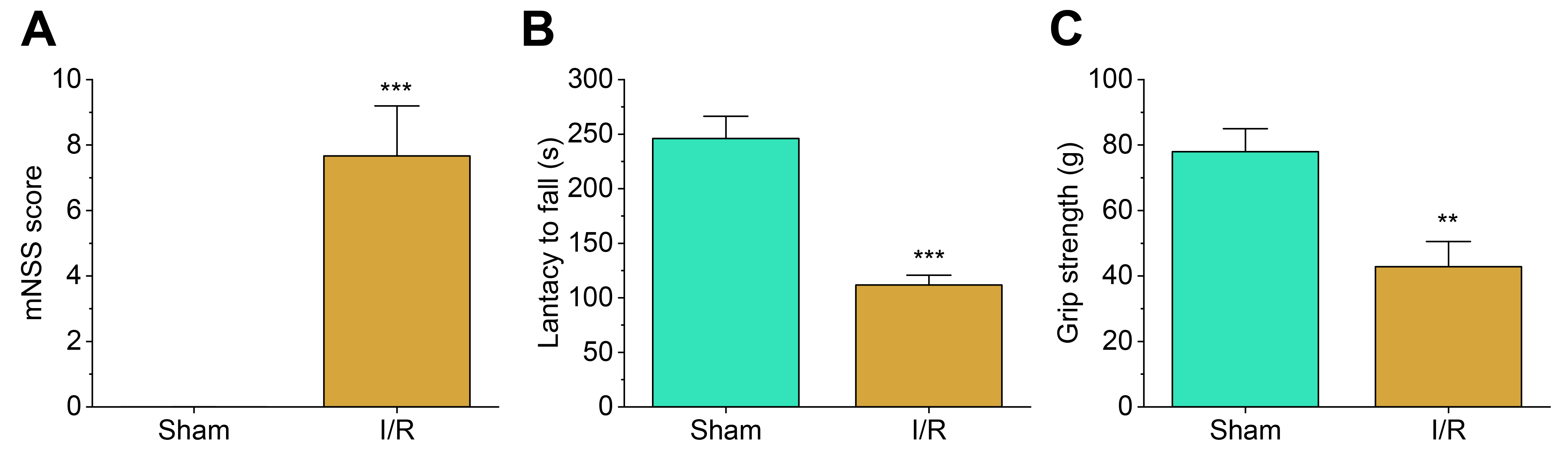


**Figure S3** Behavioral testing of I/R-injured mice. A, The mNSS of sham-operated mice and I/R-injured mice. B, The residence time of sham-operated mice and I/R-injured mice on the rotating rod. C, The maximum grip strength of sham-operated mice and I/R-injured mice. n = 3. ** *p* < 0.01, *** *p* < 0.001 *vs.* the sham group.


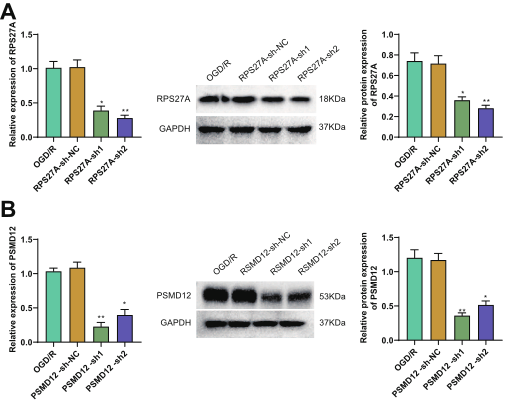


**Figure S4** In vitro validation of the silencing efficiency of the shRNA sequences. A, The RPS27A mRNA expression in microglia transfected with RPS27A-sh-NC, RPS27A-sh-1 or RPS27A-sh-2 sequence as measured by RT-qPCR and Westen Blot. B, The mRNA expression of PSMD12 in microglia transfected with PSMD12-sh-NC, PSMD12-sh-1 or PSMD12-sh-2 sequence as measured by RT-qPCR and Westen Blot. All cell experiments were repeated three times. ** *p* < 0.01 *vs.* the NC group.
